# A multicenter review of deep venous thrombosis prophylaxis practice patterns for blunt hepatic trauma

**DOI:** 10.1186/1752-2897-3-7

**Published:** 2009-06-03

**Authors:** Indraneel Datta, Chad G Ball, Lucas R Rudmik, Damian Paton-Gay, Deepak Bhayana, Peter Salat, Colin Schieman, Dean F Smith, Mary vanWijngaarden-Stephens, John B Kortbeek

**Affiliations:** 1 Department of Surgery, University of Calgary, Calgary, Canada; 2 Department of Surgery, University of Alberta, Edmonton, Canada; 3 Department of Radiology, University of Calgary, Calgary, Canada; 4 Department of Radiology, University of Alberta, Edmonton, Canada

## Abstract

**Background:**

Non-operative management of blunt hepatic trauma is successful in the majority of hemodynamically stable patients. Due to the risk of recurrent hemorrhage, pharmacologic deep venous thrombosis (DVT) prophylaxis is often delayed. The optimal timing of prophylaxis is unclear. A multi-centre, retrospective review of patients with blunt hepatic injuries presenting between 2000 and 2004 was performed. All patients had an ISS ≥ 12 and a CT scan confirming hepatic trauma. Patients were categorized into: (1) early DVT prophylaxis (≤ 48 hrs of admission), (2) delayed prophylaxis (>48 hrs), and (3) no prophylaxis.

**Methods and results:**

Thirty-seven (25%) and 45 (42%) patients received early and delayed DVT prophylaxis respectively. The remainder (32%) received none. Mean hepatic injury grades were lower in the early prophylaxis group (II) compared to the delayed and no prophylaxis cohorts (III)(p = 0.002). The number of patients requiring post-admission blood transfusions was highest in the delayed group (44%) compared to the early (26%) and no prophylaxis (6%) groups (p = 0.03). No patient in the early prophylaxis cohort developed a DVT or required delayed angiographic or operative intervention. Two patients in the delayed group failed non-operative management. Eight (18%) patients in the delayed group developed a clinically significant DVT; 1 (2%) progressed to a PE.

**Conclusion:**

Practice patterns indicate that chemical DVT prophylaxis initiated within 48 hours of admission may be safe in patients with significant blunt hepatic trauma. Delays in prevention result in venothromboembolic events, but not in fewer blood transfusions or a decreased need for subsequent angiographic or operative therapies.

## Background

Hepatic trauma complicates 25% of all blunt injuries [1]. Modern management of these patients depends primarily upon their hemodynamic stability and concurrent pattern of injury. Recent series identify that 98% of all stable patients, regardless of injury grade, can be successfully managed without an operative procedure [2].

Deep venous thrombosis (DVT) is a common complication. The observed incidence among trauma patients exceeds 50% when thromboprophylaxis is omitted [3]. Geerts and colleagues demonstrated that low molecular weight heparins (LMWH) significantly reduce the incidence of DVT compared to unfractionated heparin (UH), with rates of 7% and 15% respectively [4]. As a result, enoxaparin has become the standard pharmaceutical agent for DVT prophylaxis in multisystem trauma patients. In spite of its benefit, surgeons often delay the initiation of chemical DVT prophylaxis in those with blunt hepatic trauma due to the potential risk of recurrent bleeding. When coupled with the pathophysiology of multisystem injuries, which often include pelvic and head trauma, this delay places patients at an increased risk for DVT formation.

The optimal timing of DVT prophylaxis for blunt hepatic injured patients is unclear. The purpose of this study was to (1) identify the usage pattern of DVT prophylaxis in non-operative patients with blunt hepatic injuries among high volume trauma facilities, and (2) compare the outcomes of patients with early versus delayed initiation of chemical DVT prophylaxis.

## Methods

A multi-institutional (University of Alberta and Foothills hospitals), retrospective review of all patients with blunt hepatic injuries occurring between October, 2000 and October, 2004 was performed. The data was extracted from the province-wide Alberta Trauma Registry (ATR). This registry prospectively collects data on all patients sustaining a severe injury (Injury Severity Score (ISS) = 12) within the province of Alberta. The ATR also possesses detailed information describing the mechanism of injury, patient injuries, all therapies, and inpatient outcomes. This database has been internally validated to be 98% accurate [5].

The ethics review committees at the Universities of Calgary and Alberta approved the study protocol. Inclusion criteria included patients with an ISS ≥ 12 and a computed tomography (CT) scan confirming blunt hepatic injury. Patients were excluded if they had a penetrating liver injury, intracranial hemorrhage, or died within 24 hours of admission. They were also excluded if they underwent operative or angiographic intervention within 24 hours of admission.

Two staff radiologists, experienced in trauma imaging, completed blinded individual reviews of all patient CT imaging. Hepatic injuries were re-graded using the American Association for the Surgery of Trauma liver injury scale [6]. Both radiologists were blinded to the initial admitting radiological interpretation. Patients were then divided into three groups based on the timing of pharmacologic DVT prophylaxis. "Early" patients received DVT prophylaxis within 48 hours; "delayed" patients received prophylaxis after 48 hours; and the final group received no DVT prophylaxis during their hospital admission. DVTs were diagnosed by clinical suspicions confirmed by ultrasound imaging. Pulmonary emboli (PE) were diagnosed via thoracic CT. All compression stockings were sequential pneumatic devices. Study endpoints included post-admission blood transfusions (packed red blood cells (pRBC)), post-admission angiographic embolization, non-operative failure, DVT formation, and the rate of PE.

Statistical analysis was performed using Stata version 8.0 (Stata Corp. College Station, Texas, USA). Normally or near-normally distributed variables were reported as means and non-normally distributed variables as medians. Differences were demonstrated using ANOVA and Student t-tests. A p value less than 0.05 was considered to represent statistical significance for all comparisons.

## Results

During the study period, 390 patients were diagnosed with a hepatic injury. One hundred and six (27%) met the study inclusion criteria (Table 1). No patients died. DVT prophylaxis was administered in the early and delayed cohorts in 25% (27/106) and 42% (45/106) respectively. Thirty-four (32%) additional patients received no pharmacologic DVT prophylaxis. Demographics and initial hemodynamic measurements were similar between all groups (Table 1). There was a higher rate of blood transfusions in the delayed cohort (24%), compared to the early and no DVT prophylaxis groups (11% and 12% respectively)(p = 0.03). The mean ISS was also higher in the delayed group when compared to the no prophylaxis cohort (p = 0.02).

**Table 1:** Patient demographics and injuries

**Variable**	**Early**	**Delayed**	**None**
Total	27	45	34
Mean Age	37	42	30
Gender (% male)	63	78	59
Mean ISS	22	25	20
Mean Number of Injuries	7	9	5
Mean Admission Blood Pressure	130	124	128
Mean Admission Heart Rate	93	97	89
Blood Transfusion (%)	11	22	12
Mean Number of Blood Units	2	3	2.5
Mean Admission Hemoglobin	127	121	126
Hepatic Injury Grade:			
Mean	2.2	2.9	3.0
Grade 1	6/27 (22%)	6/45 (13%)	1/34 (3%)
Grade 2	13/27 (48%)	4/45 (9%)	9/34 (25%)
Grade 3	5/27 (19%)	24/45 (54%)	14/34 (42%)
Grade 4	3/27 (11%)	9/45 (20%)	9/34 (26%)

Grade 5	0	2/45 (4%)	1/34 (3%)

ISS = Injury Severity Score

The mean hepatic injury grades were lower in the early prophylaxis group (2.2) compared to either the delayed or no prophylaxis cohorts (2.9 and 3 respectively) (p = 0.002). Most (70%) patients in the early group were comprised of lower grade injuries (I, II). The majority of patients in the delayed group (78%) had higher injury grades (III, IV, V) (Table 1). Post-admission hemoglobin levels were stable across all cohorts (p = 0.47) (Table 2). The mean hospital length of stay (LOS) was highest in the delayed prophylaxis group (26 days), followed by the early and no prophylaxis groups (14 and 8 days respectively)(p = 0.0003) (Table 2). The delayed group also possessed the highest rate of ICU admission (44%), with an associated mean LOS of 9 days, while the no prophylaxis cohort required ICU care in only 9% of patients. Pharmacologic DVT prophylaxis was most commonly initiated on post admission day 1 for the early group and day 6 for the delayed group (Table 2). LMWH was used in 78% of patients in the early group and 84% of patients in the delayed cohort. UH was employed in the remainder.

**Table 2:** Inpatient demographics

**Variable**	**Early**	**Delayed**	**None**
Total	27	45	34
Mean Hemoglobin (PAD 2)	109	98	118
Mean Hemoglobin (PAD 5)	104	105	116
Mean Length of Hospital Stay (days)	14	26	8
ICU Admission (%)	22	44	9
Mean length of ICU Stay (days)	8	9	3
Mean PAD Ambulation Allowed	3	6	4
Use of Compression Stockings (%)	44	91	71
Mean PAD DVT Prophylaxis Started	1	6	-
LMWH (%)	78	84	-

Unfractionated Heparin (%)	22	16	-

PAD = Post Admission Day

ICU = Intensive Care Unit

DVT = Deep Venous Thrombosis

LMWH = Low Molecular Weight Heparin

Overall, the delayed group experienced the highest rate of post-admission blood transfusions (44%) when compared to both the early and no prophylaxis groups (26% and 6% respectively)(Table 3). There were no delayed arterial embolizations or non-operative management failures in the early and no prophylaxis groups. In the delayed prophylaxis group however, 2 patients failed non-operative observation. Both suffered grade 5 injuries. One of these patients also underwent pre-operative arterial embolization. No DVT or PE was diagnosed in the early or no prophylaxis groups. Eight (18%) patients were diagnosed with a DVT in the delayed group, with one (2%) patient developing a clinically significant PE.

**Table 3:** Patient outcomes

**Variable**	**Early**	**Delayed**	**None**
Total	27	45	34
Blood Transfusion Post-Admission(%)	26	44	6
Mean Number of Blood Units	2.6	4.3	1.5
Arterial Embolization (%)	0	2	0
Failure of Non-operative Therapy(%)	0	4	0
Deep Venous Thrombosis (%)	0	8/45 (18)	0
LMWH Group (%)	0	4/8 (50)	0
Pulmonary Embolus (%)	0	1/45 (2)	0

LMWH Group (%)	0	0/1 (0)	0

LMWH = Low Molecular Weight Heparin

The early and delayed prophylaxis groups were also delineated based on grade of injury (Table 4 and Table 5). There was no increase in the rates of post-admission blood transfusion, arterial embolization, or non-operative failure in patients with low grade (Grade I and II) injuries who received early pharmacologic DVT prophylaxis. There was however, 1 DVT (10%) diagnosed in a patient with a low grade injury with delayed DVT prophylaxis. Although the ISS (p = 0.53) and mean number of injuries (p = 0.26) were similar between both cohorts, the delayed group had a higher ICU admission rate (60% versus 16% respectively), and hospital LOS (24 versus 11 days respectively) (p = 0.0069).

**Table 4:** Comparison of early and delayed prophylaxis group outcomes for grade 1 and 2 hepatic injuries

**Variable**	**Early**	**Delayed**
Total	19	10
Mean ISS	21	22
Mean Length of Hospital Stay (days)	11	24
ICU Admission (%)	16	60
Mean PAD DVT Prophylaxis Started	1	6
Blood Transfusion Post-Admission(%)	26	40
Mean Number of Blood Units	2.6	4
Arterial Embolization (%)	0	0
Failure of Non-operative Therapy(%)	0	0
Deep Venous Thrombosis (%)	0	1/10 (10)

Pulmonary Embolus (%)	0	0

ISS = Injury Severity Score

ICU = Intensive Care Unit

PAD = Post Admission Day

DVT = Deep Venous Thrombosis

**Table 5:** Comparison of early and delayed prophylaxis group outcomes for grades 3, 4 and 5 hepatic injuries

**Variable**	**Early**	**Delayed**
Total	8	35
Mean ISS	26	25
Mean Length of Hospital Stay (days)	23	27
ICU Admission (%)	38	37
Mean PAD DVT Prophylaxis Started	1	6
Blood Transfusion Post-Admission(%)	25	46
Mean Number of Blood Units	2	4
Arterial Embolization (%)	0	3
Failure of Non-operative Therapy(%)	0	6
Deep Venous Thrombosis (%)	0	7/35 (20)

Pulmonary Embolus (%)	0	1/35 (3)

ISS = Injury Severity Score

ICU = Intensive Care Unit

PAD = Post Admission Day

DVT = Deep Venous Thrombosis

In patients with high grade hepatic injuries, only 8 received early pharmacologic DVT prophylaxis (Table 5). These groups had a similar ISS (p = 0.90) and mean number of injuries (p = 0.13). The ICU admission rate and hospital LOS were also similar between the early and delayed groups (p = 0.71). Two patients (grade 5 injuries) failed non-operative observation. Overall 8 (18%) DVTs and 1 (2%) PE were diagnosed in patients who had a delay in pharmacologic DVT prophylaxis.

## Discussion

Multi-system trauma is a major risk factor for the development of a DVT. In patients without thromboprophylaxis, the overall incidence exceeds 50% and the rate of proximal limb DVTs approaches 18% [3,7,8]. Although an isolated DVT is rarely life-threatening, subsequent PE often carry significant morbidity and mortality. Pulmonary embolism is estimated to be the third leading cause of death in trauma patients who survive beyond the first day [3,9-11]. While non-operative management of hepatic trauma appears to be safe in up to 98% of hemodynamically stable patients, the optimal timing of pharmacologic DVT prophylaxis is unclear [2]. There is often a delay in the administration of chemical prophylaxis in an attempt to prevent recurrent liver hemorrhage, and therefore the perceived risk of converting non-operative candidates into operative cases. As a result, physicians must balance the risk of re-bleeding with the development of a venous thromboembolism.

In this study population, only 2 patients failed non-operative observation. Both had grade 5 hepatic injuries that hemorrhaged prior to initiating DVT prophylaxis. As expected, patients with lower grade injuries were more likely to receive early prophylaxis compared to those with high grade hepatic injuries. Approximately 80% of patients with high grade injuries received delayed DVT prophylaxis. Considering the rare occurrence of non-operative treatment failure, this difference is particularly striking because only 34% of patients with low grade injuries were delayed in their chemical prophylaxis.

A significant subset of patients (33%) received no pharmacologic DVT prophylaxis. This group possessed a mean hepatic injury grade that was nearly identical to the delayed prophylaxis cohort (3 versus 2.9 respectively). They did however have a significantly shorter hospital LOS (8 versus 26 days respectively). The most likely explanation for the lack of prophylaxis in this group was that these patients were expected to receive 'delayed' prophylaxis but were discharged before chemical prevention was initiated. We view this as a treatment failure by our trauma services. These patients displayed no clinically significant DVTs or PEs during their hospital admission. We believe this is a direct result of their early ambulation. Because outpatient follow-up data was not available, the actual number of patients in any particular group who were discharged home and later developed a DVT or PE is unknown.

Neither failure in non-operative observation, nor increases in blood transfusions were observed in patients who received early DVT prophylaxis. Although limited patient numbers and an injury grade bias across study groups made it difficult to draw definitive conclusions, this study suggests that early pharmacologic DVT prophylaxis may be safe in patients with blunt liver trauma. This cannot be confirmed elsewhere in the literature because the only publication to describe a comparable patient population did not assess the use or timing of pharmacologic DVT prophylaxis [2]. It did appear that the majority of their patients who failed non-operative therapy had grade 4 and 5 injuries however [2]. It is also important to note that had our study employed prospective ultrasound screening for all trauma patients (rather than screening for those patients with a "high" clinical suspicion for DVT), the rates of DVT in the delayed and no prophylaxis cohorts may have been even more substantial.

## Conclusion

In summary, 98% of our patients underwent successful non-operative management of blunt hepatic injuries. Patients with low grade injuries generally received pharmacologic DVT prophylaxis early in their hospital admission. Most importantly, early pharmacologic DVT prophylaxis did not result in increased blood transfusion requirements or non-operative failures. This was consistent regardless of the injury grade. As a result of this data, we are now beginning to standardize early prophylaxis in our patients with blunt hepatic trauma. We believe that delays in pharmaceutical prevention place patients at a significantly increased risk for venothromboembolic events. This risk approximates 18% if prophylaxis is delayed more than 48 hours after admission. To confirm these observations, a multicenter randomized controlled trial is planned.

## Competing interests

The authors declare that they have no competing interests.

## Authors' contributions

ID – Study design, data analysis, manuscript & editing.

CGB – Data analysis, manuscript writing & editing.

LRR – Data analysis & manuscript writing.

DP-G – Data analysis.

DB – Data analysis & editing.

PS – Data analysis & manuscript writing.

CS – Data analysis & manuscript writing.

DFS – Data analysis.

MvW – Data analysis, manuscript writing & editing.

JHK – Data analysis, manuscript writing & editing.
